# Adaption and reliability of the Nutrition Environment Measures for stores (NEMS-S) instrument for use in urban areas of Chile

**DOI:** 10.1186/s12889-022-12651-w

**Published:** 2022-02-03

**Authors:** Gislaine Granfeldt, Montserrat Victoriano, Juan Antonio Carrasco, Katia Sáez, Maria del Mar Bibiloni, Josep A. Tur

**Affiliations:** 1 Department of Nutrition and Dietetics, Faculty of Pharmacy, University of Concepción, Concepción, Chile; 2 Department of Civil Engineering, Faculty of Engineering, University of Concepción, Concepción, Chile; 3 Faculty of Physical Sciences and Mathematics, Department of Statistics, University of Concepción, Concepción, Chile; 4 Research Group on Community Nutrition and Oxidative Stress, University of the Balearic Islands-IUNICS, E-07122 Palma de Mallorca, Spain; 5 Institute of Health Carlos III, CIBEROBN (Physiopathology of Obesity and Nutrition, CB12/03/30038), E-28029 Madrid, Spain; 6 Health Research Institute of the Balearic Islands (IDISBA), E-07120 Palma de Mallorca, Spain; 7 Research Group on Community Nutrition and Oxidative Stress, University of the Balearic Islands, IDISBA & CIBEROBN, Guillem Colom Bldg, Campus, E-07122 Palma de Mallorca, Spain

**Keywords:** Chile, Food availability, Food environment, Food store survey

## Abstract

**Objective:**

To adapt and assess reliability of the Chilean version of Nutritional Environment Measurement for Stores (NEMS-S-CHILE) to measure the food environment of stores in urban areas.

**Design:**

NEMS-S-CHILE was the NEMS-S tool adapted to the Chilean food patterns; foods were grouped according to level of processing in (a) unprocessed or minimally processed foods, (b) processed culinary ingredients, (c) processed foods, and (d) ultra-processed foods, and scored according to NEMS-S-CHILE tool. Reliability inter evaluators was measured.

**Setting:**

City of Concepción, Bio-Bio region, Chile.

**Participants:**

Seventeen of a total of 25 supermarkets, and 9 out of 10 street markets according to the municipal registry and the street market trade unions, representing 74.3% of both types of food premises in Concepción.

**Results:**

Reliability inter evaluators was measured by the following aspects: product availability, price, quality, and variety, through the intraclass correlation coefficient (ICC), percent agreement, and Cohen’s kappa analysis. Reliability was high for availability, where the kappa index and ICC were acceptable, ranging from moderate to high (0.42 to 1.00 for the kappa coefficient and 0.65 to 1.00 for ICC), as well as for prices (ICC: 0.65-1.00 ), variety (kappa: 0.76-1.00) and quality (percent agreement: 68.2- 100%).

**Conclusions:**

The adapted instrument, NEMS-S-CHILE, has a high reliability inter evaluators and can be useful to measure the availability of foods by the level of processing according to the prevalent food system in developing countries.

**Supplementary Information:**

The online version contains supplementary material available at 10.1186/s12889-022-12651-w.

## Introduction

The obesogenic environment, defined as “the built and dietary environment that affects patterns that lead to the accumulation of “body fat”, establishes obesity as the normal response of individuals to obesogenic environments in which they are found [1]. In the post-nutritional and demographic stage, Chile reaches the tenth place in the world among the countries with the highest obesity in adult population (74.2%). Likewise, the country reaches the fifth place among the child population [2, 3] with only 5% of the population feeding healthy [4]. The literature indicates that food consumption behavior is influenced by food environments that shape the decisions both family and individual [5–7]. These decisions affect quality and quantity of food consumed in the family diet and therefore, the intake of nutrients [8].

From the socio-ecological perspective, the study of retail food environment includes the community environment, widely studied through the density and proximity of types of premises available in the territory and the consumer environment, characterized by the availability of healthy foods, their price, and variety of options, freshness and nutritional qualities [8].

The constructs described to assess the food environment of consumers have generated many instruments based on food checklists that seek to capture environmental stimuli that can influence consumer food choices [9–11]. However, few instruments have undergone psychometric tests. The Nutritional Environment Measurement for Stores (NEMS-S) instrument [12], developed according to dietary guidelines of the North American population is aimed to collect information through the direct observation of categories and species of comparable foods in different retail stores, determining availability, quality, variety and price of the healthy food options. Its application has allowed guiding decision-making and it has been shown that has high reliability inter evaluators and apparent criterion validity [12, 13]. However, the retail food environments must be adapted to the contexts and cultural, food, ethnic and territorial contexts, and realities. Different versions of NEMS have been proposed, adapted according to representative dietary patterns of the population, adjusted to the reality of food supply chains available in each region or country [14–17]. Similarly, this is useful to observe among rural and urban areas [18, 19], ethnic populations [20] and other types of food stores [13, 16].

The retail food environment in Chile needs to be studied according to its specific characteristics. In addition to the differences in the nutritional and cultural patterns of the Chilean population, there are other differences in the distribution and sale models of food compared to developed countries. The “supermarket revolution” entered the markets strongly in the 2000’s, though neighborhood stores and temporary food markets continue to play a leading role. This group of stores called street markets are an important marketing channel of the small and medium agriculture and artisanal fishing. These street markets supply 70% of fruits and vegetables and 30% of fish, which indicates the great importance of this channel for food security and the incomes of small producers and fishermen [21].

Food systems in low and middle-income countries have experienced a growing income in the intake of ultra-processed foods. In Chile, its consumption reaches 28.6% of the total energy intake, with more than half of the total intake of added sugars. In addition, the absolute expense in ultra-processed foods has increased in 110% [22, 23]. These industrial formulations of food and beverages are produced mainly by refined substances extracted or derived from foods and additives [24, 25]. The evidence is categorical by stating that its consumption is associated with imbalances in nutrient intake and the incidence of cardiovascular diseases, displacing dietary patterns based on minimally processed or natural foods and fresh foods [26–29]. Since 2016, Chile has its own Law regulating nutritional food composition and its advertising [30].

Consequently, the objective of this study was to adapt and assess the reliability inter evaluators of the Chilean version of the Survey of Nutritional Environment Measures in (NEMS-S-CHILE) Stores in the city of Concepcion’s urban context.

## Methods

This work is part of a larger study, aimed to assess the role of the mobility of different people according to their eating patterns in the city of Concepcion’s urban context of Chile. The current research was carried out in March-May 2018 in the commune of Concepción, capital of the Bio-Bio Region, with a population of 219,057 inhabitants, and 221.6 km^2^ of area [31].

### Sample Selection

The sample selection was performed by convenience, selecting 17 out of a total of 25 supermarkets according to the information provided in the patent office of the municipality of Concepción, and 9 out of 10 street markets according to the municipal registry and the street market trade unions, representing 74.3% of both types of food premises in the commune of Concepción. The application of the instrument in convenience stores or neighborhood businesses, butchers, bakeries, or candy stores was not considered in the development of the project. The instrument was applied after the explicit authorization of the owners and/or administrators of the premises, without prior contact. A cover letter was extended to the local manager explaining the details of the study and answering any potential queries about the process. To assess reliability, inter evaluator, the instrument was applied twice on the same day.

### NEMS-S-CHILE

The original NEMS-S tool was designed to audit the retail store environment through direct observation allowing knowing the food environment of the consumers, through availability, quality, variety, and price of healthy food options in eleven food categories: milk, fruit, vegetables, ground beef, hot dogs, frozen meals, bakery, beverages, bread, potato chips and cereals. Many of these foods are not available or are not part of the cultural and food reality of other populations.

To adapt this tool to the Chilean context, a consensus was achieved in the research team made up of a sociologist, an engineer, a dietitian as well as two external dietitians who were members of the Healthy Life Center of the University of Concepción. The instrument was translated into Spanish, evaluating relevance and clarity of contents, and then adapted through three steps: (a) the selection of foods suitable to the Chilean culture, (b) the classification according to the level of food processing, and (c) adjusting a scoring system according to the availability, price, variety, and quality of food (see Supplementary material S1).

-  The food selection was performed in accordance with the Family Budget Survey [22], the National Survey of Food Consumption 2011 [4] and the dietary guidelines for the Chilean population [32, 33]. This survey represents the balance energy and identifies healthy foods for daily consumption, as well as critical nutrients for the Chilean population. The representation listed in descending order includes fruits and vegetables, dairy products, meats, legumes and eggs, grains, and oils.
- The foods were selected according to the level of industrial processing. For this purpose, the NOVA (it is not an acronym) classification by Monteiro et al. [24, 25] was applied. This classification groups foods that were classified according to the nature, scope, and purpose of their industrial processing. This classification divides foods into 4 groups:

- Group 1: Natural or minimally processed foods, which includes the removal of inedible or unwanted parts, and drying, grinding, fractionation, filtering, roasting, boiling, non-alcoholic fermentation, pasteurization, refrigeration, cooling, freezing, storage in containers, and vacuum packed.
- Group 2: Culinary ingredients, which are substances derived from Group 1 or natural foods through processes that include pressing, refining, grinding, and drying.
- Group 3: Processed foods that have two or three ingredients and are recognizable as modified versions of Group 1 foods.
- Group 4: Ultra-processed foods, including hydrogenation and hydrolyzation, extrusion and molding, as well as pre-processing for frying, in addition to the use of additives such as preservatives, antioxidants, and stabilizers.

### Score

The scoring system was modified from the original version based on the level of industrial food processing. In the original version of NEMS-S, a higher score was assigned to the healthy option low in calories, fats, and sugars. On the other hand, the NEMS-S-CHILE version where a food was considered unhealthy if it was ultra-processed, regardless of whether its composition has been modified to reduce sugar or fat content or increase fiber. The Chilean version includes 62 foods, subdivided into 27 measures, distributed in four groups according to the processing level, evaluating the availability, price, variety, and quality of the foods (Table 1). Regarding availability, Group 1 foods were considered “healthier”, Groups 2 and 3 “intermediate” and Group 4 “unhealthy”. Groups 1, 2 and 3 obtained positive points, concentrating the highest score in Group 1 for being natural or minimally processed products. Group 4 foods received negative points, setting a difference compared to the original NEMS-S which gives a positive score to ultra-processed foods low in calories, fat, and sugar.

**Table 1:** Food measurements and scoring dimensions in the Chile version of the Food Environment Measurement Survey for Stores (NEMS-CHILE)

Type of food	Availability	Quality	Comparative price	Variety
GROUP 1: Natural or minimally processed foods				
Fresh fruits	X	X		X
Fresh vegetables	X	X		X
Lean meat	X			
Chicken	X			
Fresh sea products	X			
Legumes	X			X
Oleaginous fruits	X			
Potato	X			
Rice	X		X	
Liquid milk	x		X	
GROUP 2: Processed culinary ingredients				
Oil	X		X	
Butter	X			
Wheat flour	x		X	
GROUP 3: Processed foods				
Canned seafood	X			
Yogurt	X		X	
Dried noodles	X		X	
Wholemeal bread	X		X	
Cheeses and derivatives	X		X	
Processed oleaginous fruits	X			
GROUP 4: Ultra-processed food products				
Sausages	X			
Jams	X			
Soda (carbonated drinks)	X			
Juices	X			
Processed fat	X			
Breakfast cereals	X			
Biscuits	X			
White bread	X			

*X* Score dimensions

Regarding prices, an additional score was assigned to compare monetary values. Similarly, an additional score was assigned for the same amounts of foods based on NOVA categories. The variation in the price of fruits and vegetables was not considered, due to the spatial characteristic of street markets and the difficulty in unifying the unit of measurement in the sales format.

The quality was determined for each fresh fruit and vegetable like NEMS-original. It was qualified as “adequate in quality” if more than 75% of each food reached the maximum condition according to its organoleptic characteristics (color, freshness, texture, and cleanliness). Accordingly, 3 additional points were assigned if more than 75% of the total fruits and vegetables complied with the concept of “adequate quality”, 2 points if they reached between 74% and 50% of each food, 1 point if they reached between 49% and 25% and 0 points if less than 25% met the requirement.

The variety was determined for fruits and vegetables as NEM-S, and the variety of legumes was incorporated (a food not included in the original instrument), assigning extra-scores in those premises that presented additional varieties not contemplated in the instrument. If fruits and vegetables had more than 3 additional varieties, 3 points were added to each measure. In the case of legumes, 4 additional points were assigned if there were more than 5 varieties.

Finally, scores were awarded to each food measurement, according to the previous conditions; reaching scoring ranges between -30 and 100 points compared to -8 and 50 in the original NEMS-S (see Supplementary material S2). In both versions, the highest total score per grocery store refers to the highest availability of healthy foods versus the lowest score.

### Training

The evaluators corresponded to two dietitians invited to participate in the project. They were trained to collect data, field practices, typing and scoring. They were provided with an instruction manual. Preliminary tests were carried out in four premises and minimal corrections were made to the instrument to facilitate its application, clarifying units of measurements and food formats to audit.

### Statistical analysis

All data analyses were performed with the SPSS® version 24.0 statistical program (IBM Corporation New York, NY, USA). To analyze the reliability inter evaluators in the aspects of availability, quality and variety, the percentage of general agreement and Cohen’s Kappa coefficient (k) were determined for dichotomous measures (yes/no). A Kappa values between 0 and 0.2 indicated a mild agreement, 0.21-0.40 fair agreement, 0.41-0.60 moderate agreement, 0.61-0.80 substantial agreement and between 0.81 and 1.00 perfect agreement [34]. For price comparisons, the analyses included only prices for the same type, size, and brand than that of the studied product and the intraclass correlation coefficient (ICC) was determined. An ICC value of <0.4 was considered as bad agreement, 0.41-0.59 as regular agreement, 0.60-0.74 as good agreement and >0.75 as excellent agreement [35].

## Results

The instrument was applied in 17 supermarkets and 9 street markets. These represented 74.6% of the stores of this type in the studied areas. The average application time to the instrument was 60 min for supermarkets and 80 min for street markets, due to their territorial extension.

Table 2 shows the findings for the reliability inter evaluators of food classified by group, according to the processing level. The percentage of agreement for reliability was extremely high, with a range between 84.6% and 100%. For the Kappa analysis of the 30 food items studied, 24 of them reached a perfect agreement (0.81-1.00), 4 reached a substantial agreement (0.61-0.80), and 2 obtained a moderate agreement (0.41 -0.60).

**Table 2:** Reliability measures inter evaluators in all groups of food

	Reliability inter evaluators	
**Type of food**	Kappa	**% Agreement**
GROUP 1: Natural or minimally processed foods		
Fruit fresh	0.87	96.15
vegetables fresh	1.00	100
Lean meat	0.92	96.15
Chicken	0.91	96.15
Egg	0.65	96.15
Fish	0.92	96.15
Seafood	0.84	92.31
Legumes	1.00	100
Oleaginous fruits	0.84	96.15
Potato	1.00	100
Rice	1.00	100
Milk fluid	1.00	100
GROUP 2: Processed culinary ingredients		
Oil	0.70	92.31
Butter	1.00	100
Wheatflour	1.00	100
GROUP 3: Processed foods		
Canned fish	0.70	92.31
Canned seafood	0.91	96.15
Yogurt	1.00	100
Dried noodles	1.00	100
Wholemeal bread	0.84	92.31
Cheeses and derivatives	0.42	84.62
Processed oleaginous fruits	0.43	84.62
GROUP 4: Ultra-processed food products		
Sausages	0.90	96.15
Jams	1.00	100
Soda (carbonated drinks)	1.00	100
Juices	1.00	100
Processed fat	1.00	100
Breakfast cereals	0.78	92.31
Biscuits	1.00	100
White bread	1.00	100

Reliability inter evaluators through food availability (yes/no) was examined using the kappa coefficient

Table 3 shows the reliability inter evaluators for the fresh fruits and vegetables, according to the products’ availability and quality. The percentages of agreement inter evaluators for availability (88.5-100%) and quality (75-100%) were extremely high, except for avocados, which reached a high level of agreement (68%). Kappa statistic for most fruits and vegetables was low or could not be calculated due to the high proportion of acceptable quality, or in some cases, due to lower availability of food present in stores, such as kiwi, tangerine, pear, and celery.

**Table 3:** Reliability measurements inter evaluators for product availability and quality

	Availability		Quality		
**Product**	**Kappa**	**% Agreement**	**n**	**Kappa**	**% Agreement**
FRUITS	0.87	96.2	−	−	−
Kiwi	0.91	96.20	18	0.45	88.9
Tangerine	1.00	100	18	0.47	83.3
Apple	1.00	100	21	−	100
Orange	0.78	92.3	19	−	78.9
Pear	1.00	100	18	−	100
Banana	0.88	96.2	20	−	75.0
VEGETABLES	1.00	100	−	−	−
Cerely	0.72	88.5	17	−	100
Chard	0.87	96.2	21	−	100
Lettuce	1.00	100	22	−	100
Onion	1.00	100	22	−	100
Avocado	1.00	100	22	−	68.2
Pepper	1.00	100	22	−	100
Cabbage	1.00	100	20	−	100
Tomato	1.00	100	22	−	100
Carrot	1.00	100	21	−	95.2

Consistency inter evaluators of food availability (yes/no) and food quality (yes/no) was examined through kappa coefficient. Fields with dashes indicate that statistics could not be calculated due to <2 levels/cross tabulation

Table 4 shows the reliability inter evaluators for availability, variety and price. The level of agreement for availability was extremely (80.8-100%), and the Kappa statistic showed a perfect agreement in all products analyzed, except for mature cheese. In the price, the ICC showed a high level of agreement (0.75-1.00), except for white rice, ripe cheese, and whole yogurt, which reached a good agreement (0.65-0.70). In the price comparison, the healthy options presented a smaller proportion than the regular ones, as is the case with brown rice, olive oil, whole wheat flour, whole wheat noodles, whole wheat bread, and cheese. In relation to extra variety, both percentages of agreement (92.3-100%) and kappa statistic (0.76-1.00) were exceedingly high.

**Table 4:** Reliability measurement inter evaluators for price and variety of products

	Availability ^a^		Price^b^		Variety^a^	
	**Kappa**	**% Agreement**	n	ICC	Kappa	% Agreement
**Rice**						
White	0.84	96.2	18	0.7	−	−
integral	0.91	96.2	14	1	−	−
**Liquid milk**						
Whole	1	100	18	0.99	−	−
Skim	1	100	18	1	−	−
**Oil**						
Olive	0.82	92.3	12	1	−	−
Sunflower	1	100	14	0.76	−	−
Vegetable	0.87	96.2	13	0.76	−	−
**Wheat flour**						
White	1	100	17	0.96	−	−
Integral	1	100	12	0.99	−	−
**Yogurt**						
Whole	1	100	14	0.65	−	−
Skim	1	100	16	1	−	−
**Dried noodles**						
White	0.84	96.2	21	0.75	−	−
Integral	0.84	92.3	11	0.99	−	−
**Wholemeal bread**						
Bread shake	0.92	96.2	10	0.76	−	−
Mold integral	0.84	96.2	7	0.94	−	−
**Cheese and derivatives**						
Mature cheese	0.33	80.8	15	0.70	−	−
Quesillo	0.78	92.3	11	0.89	−	−
**Other extra fruits**	−	−	−	−	0.80	92.3
**Other extra fresh vegetables**	−	−	−	−	0.76	92.3
**Other extra legumes**	−	−	−	−	1	100

*Abbreviations: ICC* intraclass correlation coefficient

^a^The consistency inter evaluators of food availability (yes/no) and the variety of food (yes / no) was examined using the kappa coefficient

^b^The degree of price consistency inter evaluators was examined by the ICC

Fields with dashes indicate that statistics could not be calculated due to <2 levels/cross tabulation

Table 5 shows the comparison between stores per food group. No significant differences were observed in Group 1 among stores. This group was composed of natural or minimally processed foods. However, the average score was higher for fruits and vegetables in street markets. In groups 2, 3 and 4 there were significant differences because supermarkets presented a greater amount of processed and ultra-processed foods. In the total score, street markets present higher average score than supermarkets.

**Table 5:** Comparison of NEMS-S-CHILE scores between food stores

	Street markets					Supermarket					
	Mean	SD	Median	Q1	Q3	Mean	SD	Median	Q1	Q3	P value
Group 1	53.6	8.6	56	50	58	52.9	17.4	62	40	62	0.385
Fruits and vegetables	26.2	4.6	28	27	28	20.5	11.9	28	11	28	0.406
Other foods G1	27.3	5.2	28	22	30	32.4	6.6	34	29	34	0.051
Group 2	1.4	2.0	1	0	2.5	7.5	0.9	8	7	8	<0.001
Group 3	4.1	3.0	5	1	6.5	12.1	1.3	13	12	13	<0.001
Group 4	3.3	3.7	2	0	6	29.4	1.4	30	29	30	<0.001
Total	55.8	9.6	59	51	62	43.2	17.7	51	31	53	0.032

*SD* Standard Deviation

## Discussion

This work constitutes the first version of a tool to measure the food environment in Chile, adapted from the original NEMS-S to Chilean context, to assess the food availability. Similarly, the eating and cultural context of the country was also considered.

NEMS-S is considered an environmental audit tool, based on observation, whose consistency is determined by reliability inter evaluators, reaching high ranges of reliability in most versions of NEMS-S, original and adapted (Brazil, China, Spain, and Canada), which clarifies that the measurement protocol and training methods are sufficient to prepare the interviewers in data collection process with high precision. For original NEMS-S, the kappa coefficient between evaluators varied from 0.83 to 1.00 [12], for the Brazilian version the kappa coefficient varied from 0.69 to 1.00 and the ICC from 0.75 to 1.00 [14]. Current NEMS-S-CHILE version showed results like NEMS-CHINA [15], where the kappa index and the ICC were acceptable, varying from moderate to high (0.42 to 1.00 for kappa coefficient and 0.65 to 1.00 for ICC).

The reliability inter evaluators measures for availability, according to the food groups studied by processing levels, were high in practically all categories evaluated. However, Group 3 of processed foods presented moderate concordance in mature cheese and nuts (0.42-0.43). A similar situation was observed in other instruments where product packaging, diversity of nutritional labels and healthy descriptors are illegible, making it difficult to understand these foods [15, 17, 36].

When analyzing the quality of food through organoleptic characteristics, mainly fruits and vegetables trained personnel are required to determine sensory parameters for the qualitative evaluation of products. In addition, in Chile, products such as fresh fruits and vegetables are mostly bought at the street market. The conditions of sale are outdoors and at room, which could generate differences in the classification of quality or freshness by limiting the choice of food. However, in this study, most of the fruits and vegetables reached a high percentage of agreement among the pollsters. The exception is the avocado (68.2%), since it is a food whose quality is subjective and highly variable because the damage mechanics, frictions and punctures that affect quality, vascular darkening, consumption maturity, and basal rot, which are not always reflected in the external appearance of the fruit [37, 38]. In relation to groups 2, 3 and 4, as the level of processing increases, the availability of these foods is greater in supermarkets than in street market.

The price difference between healthy and regular options has been studied with increasing attention in recent years [39–41]. The availability of healthy options could be influenced by the type of store, size, and rurality, and the healthy version of food can be more expensive than the less healthy or regular version.

The differentiating approach of this study is framed on the food classification according to the degree of processing, providing relevance in the score assignment to those foods in the natural or minimally processed state. This differs from the original version [12] and another adapted versions [15–17]. In this case, the focus is on the availability of healthier options, prioritizing the low intake of calories and fat, in some cases with a high degree of industrial processing. In Latin America, the eating patterns have changed dramatically, especially in recent years, influenced by the commercial opening to foreign investment, without market regulation in many cases [25]. In Chile, the supply of food to increasingly urban consumers is characterized by a high penetration of supermarket chains, decreasing the purchase in street markets [21]. This is accompanied by extensive communication and food promotion campaigns of processed food, high in levels of critical nutrients (sugar, fats, and salt). In this way, in the Latin American region, Chile and Mexico are the first consumers of ultra-processed products, consumption that is double than that of other countries in the region such as Brazil (where an adaptation of the original NEMS has already been validated. Thus, specific validation would be required, including processing level as classification.

That process has led to a shift in the eating patterns in low and middle-income countries, where the consumption of ready meals from unprocessed or minimally processed foods has changed to ultra-processed products with excessive caloric density, high in free sugars, unhealthy fats, and salt, as well as low in dietary fiber. From this perspective, the evidence is clear when establishing the risk of consuming ultra-processed foods in the increase of body weight and the appearance of cardiovascular diseases, as previous works point out [23, 27, 42].

Therefore, determining the availability of these foods could guide the association between access, availability, and epidemiological trends, as well as promoting the progress of improvement initiatives for the design of food environments in the community.

Laws 20.606 and 20.869 were established on 2012 and 2015, respectively, about the nutritional components of food and its publicity [44, 45]. It establishes a proper labeling, which is regulated by the Chilean Food Health Regulations. It must contain clear information; therefore, the population can easily understand at least regarding the content of energy, sugar, salt, and fat. It establishes the shape of the warning labeling according to the law: It is by labeling an octagonal symbol with a background black and white border, and within the text “HIGH” followed by “Saturated Fat”, “Sodium”, “Sugars” or “Calories”. Therefore, foods without warning labeling are still ultra-processed products, that is, they are made with less sugar or salt or fat, but they maintain their original formulas, characterized by the incorporation of by-products of other ingredients such as starches, protein concentrates, flavorings, stabilizers, colorants, and other components. The latter would also be associated with the NOVA classification.

Warning labeling was thought of as a strategy to improve selection, but it is not the solution to the obesity epidemic. Since changes in eating patterns must be accompanied by food education and revaluation of traditional food. In addition, strategies must be developed that facilitate access to fresh food at affordable prices and guaranteeing local production models.

A limitation that the labeling law has brought is that the industry has modified the ingredients of the food, so as not to carry logos. However, the health effects are still those attributable to ultra-processed products, which also have a high level of satiety and low cost.

Finally, regional differences in availability and prices of food have been pointed out [43]. Like previous papers of NEMS adaptations in other countries [14, 15, 20], the selected geographic area corresponds to a metropolitan sector. In this case, the city of Concepción is a representation of the retail trade and the agri-food reality of Chile. Within the commercial perimeter, where much of the trade is concentrated, and many people are mobilized, the high reliability obtained in the application of the instrument allows it to be applied in different territorial realities, and therefore to establish differences in the consumer’s food environment according to geographical location. However, since the current study was carried out in just the region of Bio-Bio, it is encouraged that further studies analyze differences between Chilean regions.

### Strengths and limitations

Regarding the strengths of the study, the interviewers corresponded to dietitians closely related to the identification of foods, varieties, and quality determination, which facilitates the application of this instrument. However, the adequate training could bring the technical skills and competencies necessary for the application of the instrument, without being a professional in the discipline. On the other hand, the instrument could be applied in supermarkets and street markets twice the same day without impediments. The kappa statistical analysis to determine the availability of a product, and the ICC to determine the level of agreement between the qualifications of the evaluators, allowed to include quality, variety, and price of food within the score. The results obtained with this methodology allowed contributing to a more complete instrument, to be applied in food stores.

The study, however, has also several limitations. First, the instrument was validated in the Province of Concepción metropolitan area, and there may be differences in the socioeconomic level and population density, which could influence the availability of food according to place. In addition, a convenience sample from supermarkets and street markets was used to measure adaptation and reliability of NEMS-S-CHILE at this stage of the project.

Second, the list of foods studied according to the level of processing, may vary depending on the seasonality and geographical distribution of the country, which implies that some foods are not available throughout the national territory. Another limitation of this work is that results cannot be extrapolated to convenience stores or especially food store environments, as these were not evaluated in this project, however, this work provides a basis for further field work. The current study was carried out in just a region of Chile, and not in extreme regions; then, the variability in availability and prices of food among regions was not evaluated, and it constitutes another limitation to be solved in future studies.

Finally, it was not possible to calculate kappa for quality due to the high proportion of acceptable quality and, in some cases, due to the low availability of food present in stores. This latter result may be influenced by the day of supply of the different places of sale, conditioned by its availability every day. However, the NEMS-S-CHILE was carried out according to the Family Budget Survey, the National Survey of Food Consumption 2011, and the Food Based Food Guidelines of Chile, which have country wide representation.

## Conclusions

The adapted instrument, NEMS-CHILE, has high reliability inter evaluators and it can be useful to measure food availability, food environment and favor health intervention actions in the Chilean context. In this study, the food classification is according to the degree of processing, which differs from the original version, seems to be more in line with the food systems of developing countries where the prevalence of chronic diseases is associated with the consumption of ultra-processed as in Chile. This study is the first adaptation in Chile to understand the consumer food environment.

## Supplementary Information

**Additional file 1.**

## Data Availability

There are restrictions on the availability of data for this trial, due to the signed consent agreements around data sharing, which only allow access to external researchers for studies following the project purposes. Requestors wishing to access the trial data used in this study can make a request to pep.tur@uib.es.

## Abbreviations

ICC: intraclass correlation coefficient
NEMS-S: The Nutritional Environment Measurement for Store
NEMS-S-CHILE: Chilean version of the Nutritional Environment Measurement for Stores
NEMS-CHINA: Chinese version of the Nutritional Environment Measurement for Stores
