# Correction to: Adaption and reliability of the Nutrition Environment Measures for stores (NEMS-S) instrument for use in urban areas of Chile

**DOI:** 10.1186/s12889-022-12768-y

**Published:** 2022-03-09

**Authors:** Gislaine Granfeldt, Montserrat Victoriano, Juan Antonio Carrasco, Katia Sáez, Maria del Mar Bibiloni, Josep A. Tur

**Affiliations:** 1 Department of Nutrition and Dietetics, Faculty of Pharmacy, University of Concepción, Concepción, Chile; 2 Department of Civil Engineering, Faculty of Engineering, University of Concepción, Concepción, Chile; 3 Faculty of Physical Sciences and Mathematics, Department of Statistics, University of Concepción, Concepción, Chile; 4 Research Group on Community Nutrition and Oxidative Stress, University of the Balearic Islands-IUNICS, E-07122 Palma de Mallorca, Spain; 5 Institute of Health Carlos III, CIBEROBN (Physiopathology of Obesity and Nutrition, CB12/03/30038), E-28029 Madrid, Spain; 6 Health Research Institute of the Balearic Islands (IDISBA), E-07120 Palma de Mallorca, Spain; 7 Research Group on Community Nutrition and Oxidative Stress, University of the Balearic Islands, IDISBA & CIBEROBN, Guillem Colom Bldg, Campus, E-07122 Palma de Mallorca, Spain

**Correction to: BMC Public Health 22, 224 (2022)**

**https://doi.org/10.1186/s12889-022-12651-w**

The original publication of this article contained several errors in the author names. The incorrect and correct author names are listed in this correction article. The original article has been updated.

**Incorrect author names**

- Granfeldt M. Gislaine
- Victoriano R. Montserrat

**Correct author names**

- Gislaine Granfeldt
- Montserrat Victoriano

