# Supplementary material for: Adaption and reliability of the Nutrition Environment Measures for stores (NEMS-S) instrument for use in urban areas of Chile
Source: BMC Public Health. 2022 Feb 3;22:224. doi: 10.1186/s12889-022-12651-w (PMC8815185; doi:10.1186/s12889-022-12651-w)
Supplement: Supplementary file 1 — Additional file 1. [file 12889_2022_12651_MOESM1_ESM.docx]

**Supplementary material S1**

| **Nutrition Environment Survey for Stores**  **NEMS-CHILE** | | | | | | | | | | | | | | | | | | | | | | | | | | | | | | | | |
| --- | --- | --- | --- | --- | --- | --- | --- | --- | --- | --- | --- | --- | --- | --- | --- | --- | --- | --- | --- | --- | --- | --- | --- | --- | --- | --- | --- | --- | --- | --- | --- | --- |
|  | | | | | | | | | | | | | | | | | | | | | | | | | | |  | |  | | | |
| **SURVEYOR IDENTIFICATION** | | | | | | | | | | | | | | | | | | | | | | | | | | | | | | | | |
|  | | | | | | | | | | | | | | | | | | | | | | | | | | |  | |  | | | |
| Name | | | | | |  | | | | | | | | | | | | | | | | | | | | | |  | |  | |  |
|  | | | | | |  | | | | | | | | | | | | | | | | | | | | | |  | |  | |  |
| Date | | | | | |  | | | | | |  | |  | | | / | |  |  | | / | |  |  |  | |  | |  | |  |
|  | | | | | | Day Month Year | | | | | | | | | | | | | | | | | | | | | |  | |  | |  |
|  | | | | | |  | | | | | | | | | | | | | | | | | | | | | |  | |  | |  |
| **STORE IDENTIFICATION** | | | | | | | | | | | | | | | | | | | | | | | | | | | | | | | | |
|  | | | | | | | | | | | | | | | | | | | | | | | | | | |  | |  | | | |
| Store name | | | | | | |  | | | | | | | | | | | | | | | | | | | | | |  | |  | |
|  | | | | | | | | | | | | | | | | | | | | | | | | | | | | |  | | | |
| Commune/City | | | | | | |  | | | | | | | | | | | | | | | | | | | | | |  | |  | |
|  | | | | | | |  | | | | | | | | | | | | | | | | | | | | | |  | |  | |
| Sector | | | | | | |  | | | | | | | | | | | | | | | | | | | | | |  | |  | |
|  | | | | | | | | | | | | | | | | | | | | | | | | | | | | | | | | |
|  | Other premises inside the supermarket | | | | | | | | | | | | | | | | | | | | | | | | | | | | | | | |
|  | Other food stores outside the supermarket | | | | | | | | | | | | | | | | | | | | | | | | | |  | |  | | | |
|  | Parking is available on site | | | | | | | | | | | | | | | | | | | | | | | | | |  | |  | | | |
|  | Paid | | | | | | | | | | | | Free | | | | | | | | | | Discount with ticket | | | | | | | | | |
|  | Presence of sidewalks | | | | | | | | | | | | | | | | | | | | | | | | | |  | |  | | | |
|  | Accessed by public transport | | | | | | | | | | | | | | | | | | | | | | | | | |  | |  | | | |
|  |  | | | | | | | | | | | | | | | | | | | | | | | | | |  | |  | | | |
|  | N° blocks from whereabouts to trade | | | | | | | | | | | | | | |  | |  | | |  | | | | | |  | |  | | | |
|  | | |  | | | | | | | | | | | | | | | | | | | | | | | |  | |  | | | |
| Opening time: | | |  |  | : | | |  | |  | | |  | | AM  PM | | | | | | | | | | | |  | |  | | | |
|  | | |  |  |  | | |  | |  | | |  | |  | | | | | | | | | | | |  | |  | | | |
| Closing time: | | |  |  | : | | |  | |  | | |  | | AM  PM | | | | | | | | | | | |  | |  | | | |
|  | | |  | | | | | | | | | | | | | | | | | | | | | | | |  | |  | | | |
| Number of cash registers: | | | | | | | | |  | |  | | |  | | | | | | | | | | | | |  | |  | | | |
|  | | | | | | | | | | | | | | | | | | | | | | | | | | |  | |  | | | |
| Comments: | |  | | | | | | | | | | | | | | | | | | | | | | | | |  | |  | | | |
|  | |  | | | | | | | | | | | | | | | | | | | | | | | | |  | |  | | | |

| **NEMS-CHILE**  **GROUP #1 NATURAL OR MINIMALY PROCESSED FOODS** |
| --- |

| 1. **Are there fresh fruit and** **vegetables?** Yes No | **If yes, continue. If no,**  **move on to the next group.** |
| --- | --- |

|  | **Available** | | **Quality** | | | |
| --- | --- | --- | --- | --- | --- | --- |
|  |  | | Level of acceptability | | | |
|  |  | | > 25 | 25-49 | 50-75 | <75 |
| 1.Banana | Yes | No |  |  |  |  |
| *Varieties available: N° ______________* | | | | | | |
|  |  |  |  |  |  |  |
| 2. Apple | Yes | No |  |  |  |  |
| *Varieties available: N° ______________* | | | | | | |
|  |  |  |  |  |  |  |
| 3. Orange | Yes | No |  |  |  |  |
| *Varieties available: N° ______________* | | | | | | |
|  |  |  |  |  |  |  |
| 4. Pear | Yes | No |  |  |  |  |
| *Varieties available: N° ______________* | | | | | | |
|  |  |  |  |  |  |  |
| 5. Kiwi | Yes | No |  |  |  |  |
| *Varieties available: N° ______________* | | | | | | |
|  |  |  |  |  |  |  |
| 6. Tangerine | Yes | No |  |  |  |  |
| *Varieties available: N° ______________* | | | | | | |
|  |  |  |  |  |  |  |
| Other fresh fruits not mentioned | | Yes | No | How many? | |  |

|  |  |  |  |  |  |  |
| --- | --- | --- | --- | --- | --- | --- |
| 1.Tomato | Yes | No |  |  |  |  |
| *Varieties available: N° ______________* | | | | | | |
|  |  |  |  |  |  |  |
| 2.Lettuce | Yes | No |  |  |  |  |
| *Varieties available: N° ______________* | | | | | | |
|  |  |  |  |  |  |  |
| 3. Chard | Yes | No |  |  |  |  |
| *Varieties available: N° ______________* | | | | | | |
|  |  |  |  |  |  |  |
| 4. Cabbage | Yes | No |  |  |  |  |
| *Varieties available: N° ______________* | | | | | | |
|  |  |  |  |  |  |  |
| 5. Onion | Yes | No |  |  |  |  |
| *Varieties available: N° ______________* | | | | | | |
|  |  |  |  |  |  |  |
| 6. Bell pepper | Yes | No |  |  |  |  |
| *Varieties available: N° ______________* | | | | | | |
|  |  |  |  |  |  |  |
| 7. Celery | Yes | No |  |  |  |  |
| *Varieties available: N°_______* | | | | | | |
|  |  |  |  |  |  |  |
| 8. Avocado | Yes | No |  |  |  |  |
| *Varieties available: N° ______________* | | | | | | |
|  |  |  |  |  |  |  |
| 9. Carrot | Yes | No |  |  |  |  |
|  | | | | | | |
| Other fresh vegetables | | Yes | No | How many? | |  |

| 1. **Are there** **other natural foods??** Yes No | **If yes, continue. If it's No,**  **move on to the next group.** |
| --- | --- |

|  | **Available** | |  | **Variety** | | | **Price** | | |
| --- | --- | --- | --- | --- | --- | --- | --- | --- | --- |
|  |  | |  | 1-2 | 3-4 | >5 |  |  | |
| 1.Lean meat | Yes | No |  |  |  |  |  |  |  |
| *Low fat beef* | | |  |  |  |  |  |  | |
|  |  |  |  |  |  |  |  |  |  |
| 2. Chicken | Yes | No |  |  |  |  |  |  |  |
| *Chicken breast* | | | |  |  |  |  |  | |
|  |  |  |  |  |  |  |  |  |  |
| 3. Egg | Yes | No |  |  |  |  |  |  |  |
|  | | | |  |  |  |  |  | |
|  |  |  |  |  |  |  |  |  |  |
| 4. Fresh seafood | Yes | No |  |  |  |  |  |  |  |
|  |  |  |  |  |  |  |  |  | |
|  |  |  |  |  |  |  |  |  | |
| 5. Fresh fish | Yes | No |  |  |  |  |  |  | |
|  |  |  |  |  |  |  |  |  | |
|  |  |  |  |  |  |  |  |  |  |
| 6. Legumes | Yes | No |  |  |  |  |  |  |  |
| *Beans, lentils, chickpeas, peas, dried beans, etc.* | | | |  |  |  |  |  | |
|  |  |  |  |  |  |  |  |  |  |
| 7. Oleaginous fruit | Yes | No |  |  |  |  |  |  |  |
| *Nuts, almonds, pistachios, etc. (not processed)* | | | | | | | | | |
|  |  |  |  |  |  |  |  |  |  |
| 8. Potato | Yes | No |  |  |  |  |  |  |  |
|  |  |  |  |  |  |  |  |  |  |
| 9. Rice | Yes | No |  |  |  |  | $ |  | 1 kg |
|  |  |  |  |  |  |  |  |  |  |
| 10. Brown rice | Yes | No |  |  |  |  | $ |  | 1 kg |
|  |  |  |  |  |  |  |  |  |  |
| 11. Whole milk | Yes | No |  |  |  |  | $ |  | 1 L |
|  |  |  |  |  |  |  |  |  |  |
| 12. Low fat milk | Yes | No |  |  |  |  | $ |  | 1 L |
|  |  |  |  |  |  |  |  |  |  |
| 13. Skimmed milk | Yes | No |  |  |  |  | $ |  | 1 L |

| **NEMS-CHILE**  **GROUP #2** **PROCESSED CULINARY FOODS** |
| --- |

| 1. **Are there culinary ingredients??** Yes No | **If yes, continue. If it's No,**  **move on to the next group.** |
| --- | --- |

|  | **Available** | |  | **Price** | | |
| --- | --- | --- | --- | --- | --- | --- |
|  |  | |  |  |  | |
|  |  |  |  |  |  |  |
| 1. Vegetable oil | Yes | No |  | $ |  | 1 L |
|  |  |  |  |  |  |  |
| 2. Sunflower oil | Yes | No |  | $ |  | 1 L |
|  |  |  |  |  |  |  |
| 3. Olive oil | Yes | No |  | $ |  | 1 L |
|  |  |  |  |  |  |  |
| 4. Butter | Yes | No |  |  |  |  |
|  |  |  |  |  |  |  |
| 5. Wheat flour | Yes | No |  | $ |  | 1 kg |
|  |  |  |  |  |  |  |
| 6. Whole wheat flour | Yes | No |  | $ |  | 1 kg |
|  |  |  |  |  |  |  |

| **NEMS-CHILE**  **GROUP #3** **PROCESSED FOODS** |
| --- |

| 1. **Are there processed foods??** Yes No | **If yes, continue. If no,**  **move on to the next group.** |
| --- | --- |

|  | **Available** | |  | **Price** | | |
| --- | --- | --- | --- | --- | --- | --- |
|  |  | |  |  |  | |
|  |  |  |  |  |  |  |
| 1. Canned fish | Yes | No |  |  |  |  |
|  |  |  |  |  |  |  |
| 2. Canned seafood | Yes | No |  |  |  |  |
|  |  |  |  |  |  |  |
| 3. Whole beaten yogurt | Yes | No |  | $ |  | 125 g |
|  |  |  |  |  |  |  |
| 4. Low sugar and fat yogurt | Yes | No |  | $ |  | 125 g |
|  |  |  |  |  |  |  |
| 5. Dried noodles | Yes | No |  | $ |  | 400 g |
|  |  |  |  |  |  |  |
| 6. Whole grain dried noodles | Yes | No |  | $ |  | 400 g |
|  |  |  |  |  |  |  |
| 7. Wholemeal bread mold | Yes | No |  | $ |  | 500 g |
|  |  |  |  |  |  |  |
| 8. Whole Bread | Yes | No |  | $ |  | 500 g |
|  |  |  |  |  |  |  |
| 9. Mature cheese | Yes | No |  | $ |  | 250 g |
|  |  |  |  |  |  |  |
| 10. Fresh cheese | Yes | No |  | $ |  | 250 g |
|  |  |  |  |  |  |  |
| 11. Processed oleaginous fruit | Yes | No |  |  |  |  |
|  |  |  |  |  |  |  |

| **NEMS-CHILE**  **GROUP #4** **ULTRA-PROCESSED FOODS** |
| --- |

| 1. **Are there ultra-processed foods?** Yes No | **If yes, continue. If no,**  **move on to the next group.** |
| --- | --- |

|  |  | **Available** | |
| --- | --- | --- | --- |
|  |  |  | |
|  |  |  |  |
| 1. Frankfurter |  | Yes | No |
|  |  |  |  |
| 2. Sausages |  | Yes | No |
|  |  |  |  |
| 3. Burgers |  | Yes | No |
|  |  |  |  |
| 4. Cold cuts |  | Yes | No |
|  |  |  |  |
| 5. Turkey ham |  | Yes | No |
|  |  |  |  |
| 6. Sugar jams |  | Yes | No |
|  |  |  |  |
| 7. Sugar-free jams |  | Yes | No |
|  |  |  |  |
| 8. Sugar sodas |  | Yes | No |
|  |  |  |  |
| 9. Sugar-free sodas |  | Yes | No |
|  |  |  |  |
| 10. Liquid juices with sugar |  | Yes | No |
|  |  |  |  |
| 11. Sugar-free liquid juices |  | Yes | No |
|  |  |  |  |
| 12. Margarine |  | Yes | No |
|  |  |  |  |
| 13. Breakfast cereals with sugar |  | Yes | No |
|  |  |  |  |
| 14. Low-sugar breakfast cereals |  | Yes | No |
|  |  |  |  |
| 15. High sugar and fat biscuits |  | Yes | No |
|  |  |  |  |
| 16. Low sugar and fat biscuits |  | Yes | No |
|  |  |  |  |
| 17. White sliced bread |  | Yes | No |
|  |  |  |  |
| 18. Bread hallulla |  | Yes | No |
|  |  |  |  |

**Supplementary material S2: Scoring ranges in NEMS-Chile.**

| GROUP #1: Natural or minimally processed foods | | | | | | |
| --- | --- | --- | --- | --- | --- | --- |
|  | Availability | Points | Price | Points | Quality suitable | Points |
| Product not present | | 0 |  |  |  |  |
| Fresh fruit | 1-2 varieties | 2 |  |  | <25% | 0 |
|  | 3-4 varieties | 4 |  |  | 25-49% | 1 |
|  | 5-6 varieties | 6 |  |  | 50-74% | 2 |
|  | > 6 varieties | 8 |  |  | >75% | 3 |
| If there are 3 or more varieties not mentioned | | 3 |  |  |  |  |
| Fresh vegetables | 1-3 varieties | 2 |  |  | <25% | 0 |
|  | 4-6 varieties | 4 |  |  | 25-49% | 1 |
|  | 7-9 varieties | 6 |  |  | 50-74% | 2 |
|  | >9 varieties | 8 |  |  | >75% | 3 |
| If there are 3 or more varieties not mentioned | | 3 |  |  |  |  |
| Lean meat | Low fat beef | 4 |  |  |  |  |
| Chicken | Chicken breast | 4 |  |  |  |  |
| Egg | Egg | 4 |  |  |  |  |
| Fresh sea products | Fresh fish | 4 |  |  |  |  |
|  | Fresh seafood | 4 |  |  |  |  |
| Legumes  (beans, lentils, peas, chickpeas, dried beans, etc.) | 1-2 varieties | 1 |  |  |  |  |
|  | 3-4 varieties | 2 |  |  |  |  |
|  | >5 varieties | 4 |  |  |  |  |
| Oleaginous fruits | Not processed | 4 |  |  |  |  |
| Potato | Potato | 4 |  |  |  |  |
| Rice | Whole grain | 4 | Integral ≤ white rice | 2 |  |  |
|  | White | 1 |  |  |  |  |
| Milk | Skimmed | 4 | Skimmed ≤ whole milk | 2 |  |  |
|  | Whole | 1 |  |  |  |  |

| GROUP #2: Processed culinary ingredients | | | | | | | | |
| --- | --- | --- | --- | --- | --- | --- | --- | --- |
|  | | Availability | Points | Price | Points | Suitable quality | Point |  |
| Product not present | | | 0 |  |  | | |  |
| Oil | Olive | | 3 |  |  |  | |  |
|  | Sunflower | | 2 | Sunflower ≤ vegetable | 2 |  |  |  |
|  | Vegetable | | 1 |  |  |  |  |  |
| Butter | Butter | | 3 |  |  |  |  |  |
| Wheat flour | whole wheat flour | | 3 | whole wheat flour ≤  wheat flour | 2 |  |  |  |
|  | Wheat flour | | 1 |  |  |  |  |  |

| GROUP #3: Processed foods | | | | | | |
| --- | --- | --- | --- | --- | --- | --- |
|  | Availability | Points | Price | Points | Quality suitable | Point |
| Product that is not present | | 0 |  |  | |  |
| Canned seafood | Canned fish | 1 |  |  |  |  |
|  | Canned seafood | 1 |  |  |  |  |
| Yogurt | Low sugar and fat | 2 | Low ≤ a Whole | 1 |  |  |
|  | Whole beaten | 1 |  |  |  |  |
| Dried noodles | Whole grain | 2 | Whole grain ≤ white | 1 |  |  |
|  | White | 1 |  |  |  |  |
| Wholemeal bread | Mold | 2 | Mold wholemeal ≤ bread shake | 1 |  |  |
|  | Beaten bread | 1 |  |  |  |  |
| Cheeses | Fresh cheese | 2 | Fresh cheese ≤ a mature cheese | 1 |  |  |
|  | Mature cheese | 1 |  |  |  |  |
| Processed oleaginous fruits | Processed oleaginous fruits | 1 |  |  |  |  |

| GROUP #4: Ultra processed food products | | | | | | | |
| --- | --- | --- | --- | --- | --- | --- | --- |
|  | Availability | Point | Price | Points | Quality suitable | | Point |
| Product that is not present | | 0 |  |  |  |  | |
| Sausages | Frankfurter | -2 |  |  |  |  | |
|  | Sausages | -2 |  |  |  |  | |
|  | Burgers | -2 |  |  |  |  | |
|  | Cold cuts | -2 |  |  |  |  | |
|  | Turkey jam | -1 |  |  |  |  | |
| Jams | Sugar free | -1 |  |  |  |  | |
|  | With sugar | -2 |  |  |  |  | |
| Soda (carbonated drinks) | Sugar free | -1 |  |  |  |  | |
|  | With sugar | -2 |  |  |  |  | |
| Juices | Low sugar | -1 |  |  |  |  | |
|  | With sugar | -2 |  |  |  |  | |
| Processed fat | Margarine | -2 |  |  |  |  | |
| Breakfast cereals | Low sugar | -1 |  |  |  |  | |
|  | With sugar | -2 |  |  |  |  | |
| Biscuits | Low sugar and fat | -1 |  |  |  |  | |
|  | High sugar and fat | -2 |  |  |  |  | |
| White bread | Mold | -2 |  |  |  |  | |
|  | Hallulla | -2 |  |  |  |  | |

Scoring ranges: subtotal availability: -30 to 72; subtotal price: 0-12; subtotal quality: 0-6; Subtotal variety: 0-10. Total NEMS-CHILE score range: -30 to 100.
